# Case Report: Neurological adverse events in subject with myasthenia gravis after PCSK9 inhibitor administration

**DOI:** 10.3389/fcvm.2024.1343775

**Published:** 2024-03-12

**Authors:** Věra Adámková, Martina Vitásková, Jaroslav A. Hubáček

**Affiliations:** ^1^ Department of Preventive Cardiology, Institute for Clinical and Experimental Medicine, Prague, Czechia; ^2^ Department of Nephrology, 1st Faculty of Medicine, Charles University, Prague, Czechia; ^3^ Faculty of Biomedical Engineering, Czech Technical University, Prague, Czechia; ^4^ Experimental Medicine Centre, Institute for Clinical and Experimental Medicine, Prague, Czechia; ^5^ Third Department of Internal Medicine, 1st Faculty of Medicine, Charles University, Prague, Czechia

**Keywords:** PCSK9 inhibitor, myasthenia gravis, undesirable side effect, treatment, cholesterol

## Abstract

**Background:**

Myasthenia gravis is a rare chronic autoimmune neuromuscular disorder mainly caused by autoantibodies to the nicotinic acetylcholine receptor. Cholesterol is an essential molecule that affects the distribution and proper functioning of this receptor. Several reports have described the potential worsening of myasthenia gravis in patients treated with statins.

**Case presentation:**

The patient was an obese 72 years old man, past smoker, diagnosed with ischaemic heart disease, type 2 diabetes mellitus and lipid metabolism disorder. Statin treatment was not implemented because of chronic myasthenia gravis and PCSK9i monotherapy [Repatha (evolucamab), 140 mg] was implemented to treat dyslipidaemia. Within 24 h after the first dose of PCSK9i the patient developed severe muscle weakness, joint pain, fever, and general discomfort, lasting for several days. Despite strong advice against the second dose administration, this was self-administered approximately 2 weeks later, leading to report significant worsening of the muscle problems, leading to the patient admittion to the neurology department where he was being treated for myasthenia gravis attack.

**Conclusion:**

Based on the neurologist's conclusion, it can be assumed that in this case, treatment with PCSK9i resulted in significant worsening of the patient's chronic disease.

## Introduction

Disturbances in lipid metabolism are a serious risk factor for the development of atherosclerotic cardiovascular disease (1). HMGCoA reductase inhibitors (statins) are widely used and represent the gold standard in the treatment of dyslipidaemia (2). However, in patients with severe hypercholesterolemia, they are not always successful in achieving the targets recommended in the guidelines proposed by professional societies (3). In such cases, PCSK9 inhibitors (PCSK9i) are new and effective drugs for treating dyslipidaemia (2).

PCSK9 (proprotein convertase subtilisin/kexin type 9) is a proteolytic enzyme that indirectly reduces the number of LDL particles in the plasma (4). PCSK9 binds to the LDL receptor/LDL particle complex and prevents LDL receptor recycling. Because PCSK9 mediates premature LDL receptor degradation in the liver, inhibition of PCSK9 activity by monoclonal antibodies reduces LDL receptor degradation and consequently increases plasma LDL cholesterol clearance (5).

Treatment of dyslipidaemia with statins occasionally leads to muscle symptoms such as myalgia/myopathy and, rarely, to severe rhabdomyolysis (6, 7) (approximately 1‰ of statin users), which is thought to be partly genetically determined (8). Treatment with PCSK9 inhibitors (PCSK9i) has a more favourable safety profile and is better tolerated than treatment with statins according to the findings in randomised trials (9). Real-world data mostly only show acute side effects, such as injection site reactions, influenza-like illness and mild myalgia, occurring in approximately 10% of patients (10). The only current contraindication to treatment with PCSK9i (according to the SPC) is allergy to any substances contained in the drug.

## Case presentation

We report the case of a possible association between treatment with PCSK9i and the occurrence of adverse neurological outcomes in a subject with a previous diagnosis of myasthenia gravis.

The patient was an obese (BMI 30.8 kg/m^2^) man who was 72 years old and who underwent thymoma resection in 2005, urgent examination in 2007 (IKEM), which revealed weakness, fainting, and unremarkable internal and cardiac findings. He was diagnosed with myasthenia gravis (nonspecified immunosuppressive therapy was administered during long stay in Moscow) in 2010. He was diagnosed with ischaemic heart disease in 2015 and prescribed aspirin 100 mg; PCI (2× DES) was performed. The patient suffered from type 2 diabetes mellitus (treated with Glucophage 850 mg), arterial hypertension (Valsacor 80 mg, Orcal neo 5 mg) and lipid metabolism disorder. Total cholesterols ranged from 5.80 to 6.30 mmol/L. He stopped smoking 15 years prior, and reported having an allergy to penicillin.

In 2022, elevated lipid values was observed (TC—5.95 mmol/L, LDL-C—4.35 mmol/L, HDL-C—0.95 mmol/L and TG—2.90 mmol/L); postprandial glycaemia 9.7 mmol/L; additional biochemical parameters (Na^+^; K^+^; Cl^−^; bilirubin; AST; ALTl; urea; S-creatinine; hs-troponin Tb; blood counts and CRP) were in the normal range. ECG—sinus rhythm, rare ventricular extra systoles. Statin treatment was not implemented because of chronic myasthenia gravis (as a contraindication) and the patient's strict negative attitude towards statin treatment.

After considering the indication criteria, PCSK9i monotherapy was implemented to treat dyslipidaemia.

According to the patient′s family (the patient did not personally contact the doctor), within 24 h after the first dose of PCSK9i [Repatha (evolucamab), 140 mg], the patient developed severe muscle weakness, joint pain, fever, and general discomfort, lasting for several days. The PCR test for SARS-CoV-2 positivity was negative.

The physician strongly advised against the administration of the second dose of PCSK9i and to contact the treating physician immediately. Despite this fact, the second identical dose was self-administered approximately 2 weeks later, and the family called again to report significant worsening of the muscle problems within 24 h after PCSK9i administration, leading to the patient being admitted to the neurology department where he was being treated for myasthenia gravis. The patient was unable to squat, had impaired gait stability and speech impairments. Magnetic resonance of the nervous system reveal no abnormalities. Patient has been treated by corticosteroids (10 mg/day) but without any improvement. Finally, pregabalin has been administered and patient was subsequently treated with five plasmaphereses, leading to the slow improvements of muscle problems.

Electromyographic findings were as follows: n. medianus DML 120%, lowering of the amplitude of CMAP (muscle action potential), n. ulnaris DML 120%, n. perineus l.sin. DML, 120%, F waves increased by more than 130%.

The finding is indicative of subacute demyelinating axonal, sensorimotor polyneuropathy of the lower extremities. Changes in more than two nerves support dg. autoimmune polyradiculoneuropathy/suspicious acute inflammatory demyelinating polyneuropathy. Based on the neurologist's conclusion, it can be assumed that in this case, treatment with PCSK9i resulted in significant worsening of the patient's chronic disease.

## Discussion and literature review

The patient was at very high risk of cardiovascular complications (heart ischaemic disease). The recommended value of LDL cholesterol is below 1.4 mmol/L. LDL cholesterol remains the basic treatment goal event in the secondary prevention of HCD (the principle “the lower the better”). The modern trends of the recommendations postulate a requirement not only for achievement a certain target concentrations of LDL-c, but also the need for a minimum 50% reduction compared to the level before treatment.

Myasthenia gravis (MG) is a rare (prevalence ∼1:5,000) chronic autoimmune neuromuscular disorder mainly caused by autoantibodies to the nicotinic acetylcholine receptor (nAChR) (11). It is partially genetically determined, as signals within acetylcholine receptor subunits alpha 1 and beta1 were recently detected using a GWAS screening (12) to be associated with MG.

Cholesterol is an essential molecule that affects the distribution and proper functioning of this receptor in both the peripheral and central nervous systems [reviewed by Paz and Barrantes (13)]. It has also been described that inhibition of cholesterol synthesis can affect nAChR trafficking to the cell surface (14).

In view of the abovementioned facts, it is not surprising that several reports have described the potential worsening of MG and the induction of myotoxicity in these patients receiving treatment with statins (15, 16). Furthermore, the description of several cases illustrates that statins may act as unmasking agents in subjects with latent neuromuscular disorders (17).

Recent results from large Mendelian randomisation (MR) studies examining drug targets contradict the generalisation of the relationship between lipid-lowering drugs and MG (18). Analyses have been focused on the role of PCSK9 inhibitors and statins on increased risk of autoimmune diseases. Generally, PCSK9i seems to be more favourable than statins. Using data from almost 40,000 subjects, lower activity of PCSK9 was not associated with a significantly increased risk of myasthenia gravis.

There are several factors that could (in the addition to the expected strong and sudden depletion of cholesterol) facilitate the worsening of neurological problems in our case. First, it could be increased age as well as obesity, both of which coincide with low physical activity, which is associated with MG (19). The fact that subjects underwent thymoma resection could also play an important role (20). Finally, although Mendelian randomisation excludes the role of lower PCSK9 activity in MG development, it needs to be mentioned that genetic variants, serving in MR as a proxy for lower activity of PCSK9, have a far smaller effect on PCSK9 activity in comparison to PCSK9 inhibitors.

A significant worsening of myasthenia gravis after the use of PCSK9 inhibitors, as in our case, has not yet been reported in the literature.

It is worth mentioning, that a neurological complication, namely peripheral neuropathy has been described in subjects with prediabetes after application of PCSK9i (21). The role of PCSK9 and PCSK9 inhibition in the peripheral nervous system has been recently summarised (22). In non-mammalian models, knocking down the PCSK9 results in defective neurogenesis and is lethal (23). In another model (mice), inhibition of PCSK9 attenuates neuronal apoptosis (24). In humans, however, brain/neuronal expression is low, and circulating PCSK9 has not the ability to cross the blood-brain barrier. Blood-nerve barrier is in contrast permeable for PCSK9 (25), making the peripheral nervous system potentially susceptible to PCSK9 inhibition.

It seems that in some cases, myasthenia gravis could be a potential limitation of PCSK9i treatment, both in primary and secondary prevention of cardiovascular disease. In such rare cases, where pharmacological treatment of hypercholesterolemia is not possible for different reasons, the last suggested option is LDL apheresis (26). However, this treatment is time-consuming and is subject to special approval.

## Limitations

Major limitation of the study is the lack of details about the immunosuppressive therapy, as the disease has been diagnosed and treated in abroad.

## Conclusion

Our report suggests that although PCSK9i treatment seems not to be associated with new-onset myasthenia gravis in the general population, it could, under some circumstances, potentially lead to worsening problems in subjects with confirmed myasthenia gravis. In conclusion, especially in polymorbid elderly subjects with MG, thoughtful clinical examination should precede treatment with PCSK9i and it should be carefully monitored.

## Data Availability

The datasets presented in this article are not readily available because all available data are within the manuscript, nothing else is known. Requests to access the datasets should be directed to vead@ikem.cz.
